# Potential Threats Posed by New or Emerging Marine Biotoxins in UK Waters and Examination of Detection Methodologies Used for Their Control: Cyclic Imines

**DOI:** 10.3390/md13127057

**Published:** 2015-11-26

**Authors:** Keith Davidson, Clothilde Baker, Cowan Higgins, Wendy Higman, Sarah Swan, Andrea Veszelovszki, Andrew D. Turner

**Affiliations:** 1 Scottish Association for Marine Science, Scottish Marine Institute, Oban PA37 1QA, Scotland, UK; scs@sams.ac.uk (S.S.); veszi@yahoo.com (A.V.); 2 Centre for Environment Fisheries and Aquaculture Science (Cefas), Barrack Road, The Nothe, Weymouth, Dorset DT4 8UB, UK; clothilde.baker@campdenbri.co.uk (C.B.); wendy.higman@cefas.co.uk (W.H.); Andrew.turner@cefas.co.uk (A.D.T.); 3 Agri-food and Biosciences Institute (AFBI), Newforge Lane, Belfast BT9 5PX, Northern Ireland, UK; cowan_higgins@msn.com

**Keywords:** cyclic imines, shellfish, harmful phytoplankton, biotoxins

## Abstract

Cyclic imines (CIs) are a group of phytoplankton produced toxins related to shellfish food products, some of which are already present in UK and European waters. Their risk to shellfish consumers is poorly understood, as while no human intoxication has been definitively related to this group, their fast acting toxicity following intraperitoneal injection in mice has led to concern over their human health implications. A request was therefore made by UK food safety authorities to examine these toxins more closely to aid possible management strategies. Of the CI producers only the spirolide producer *Alexandrium ostenfeldii* is known to exist in UK waters at present but trends in climate change may lead to increased risk from other organisms/CI toxins currently present elsewhere in Europe and in similar environments worldwide. This paper reviews evidence concerning the prevalence of CIs and CI-producing phytoplankton, together with testing methodologies. Chemical, biological and biomolecular methods are reviewed, including recommendations for further work to enable effective testing. Although the focus here is on the UK, from a strategic standpoint many of the topics discussed will also be of interest in other parts of the world since new and emerging marine biotoxins are of global concern.

## 1. Introduction

The cyclic imines (CIs) are a heterogeneous group of marine natural products with common macrocyclic features and an active imine moiety [1]. Within the CIs group, there are a number of sub-groups including the spirolides (SPXs) [2,3], the gymnodimines (GYMs) [4], the pinnatoxins (PnTXs) [5], the pteriatoxins (PtTX) [6], the prorocentrolides (PcTXs) [7,8], the spiro-prorocentrimines [9] and portimine [10]. The members of the CI toxin group show a high degree of structural similarity and are soluble in organic solvents. CIs are sometimes referred to as “fast-acting toxins” as they are characterised by acute toxicity in mice following intraperitoneal (i.p.) injection and rapid onset of neurological symptoms potentially leading to death if the compounds are present in sufficient concentrations. No human intoxications have unequivocally been linked to CIs [11] and hence the concentration of CIs in shellfish is not regulated in the European Union or in other parts of the world. However, the European Union Reference Laboratory (EURL) working group on toxicology proposed a guidance level of 400 µg SPXs/kg of shellfish [12,13]. Hence, given their potential impact on human health, CIs were included in a review of biotoxin risks in UK waters commissioned by the UK food safety authorities, the results of which are presented here.

### 1.1. Spirolides

The SPX group is the largest and best characterized CIs sub-group with currently 16 SPX analogues isolated; its compounds having been detected in European and North American waters. The marine dinoflagellate *Alexandrium ostenfeldii* was first identified as the causative organism of spirolide shellfish toxins [14]. More recently, *Alexandrium peruvianum* has also been identified as a SPXs producer [15].

Six major congeners, SPX-A, B, C, D, E and F, were initially isolated from lipophilic extracts of the digestive gland of mussels (*Mytilus edulis*) and scallops (*Placopecten magellanicus*) harvested from aquaculture sites in Nova Scotia, Canada [2,3], with spirolide toxin profile varying significantly with geographical location [14]. Subsequently, SPX-G was isolated from a culture of *A. ostenfeldii* from Denmark [16]. More recently, SPX-H and I have been added to the SPXs [17]. Like other marine biotoxins, the SPXs can be metabolised in shellfish to fatty acid esters [18,19].

**Figure 1:**
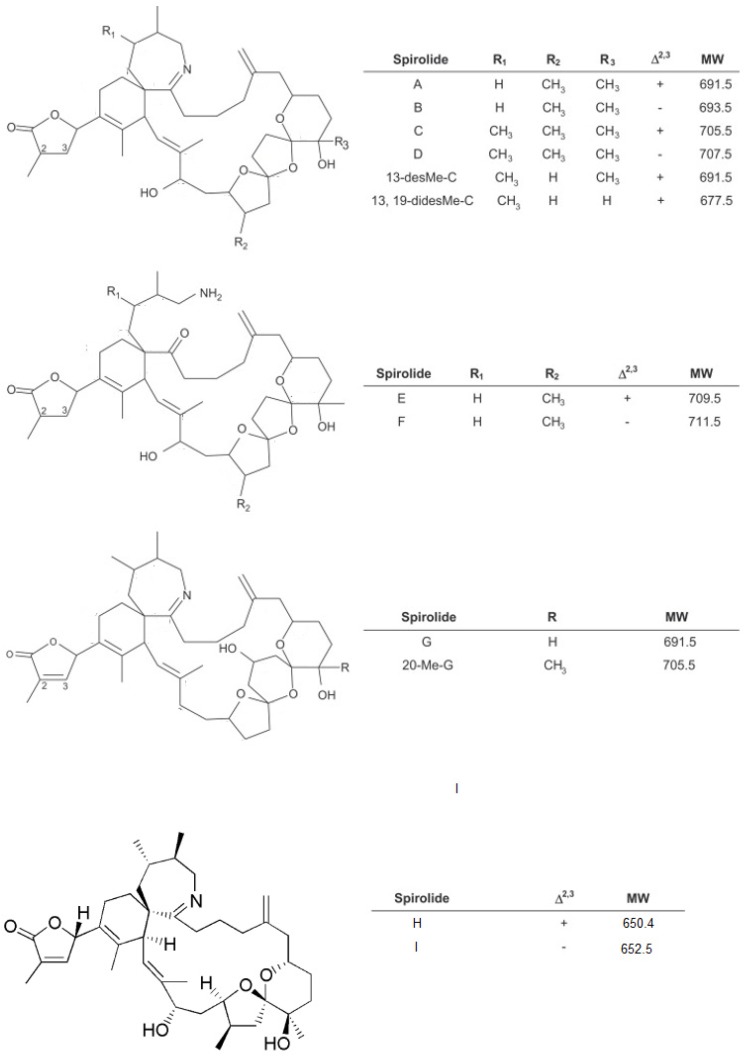
Structure of some of the known spirolides [17,20].

Until recently, SPXs were characterised by a spiro-linked tricyclic ether ring system and an unusual seven-membered spiro-linked cyclic iminium moiety, but a spirolide subclass including two compounds displaying a spiro-linked dicyclic ether ring system has now been proposed [17]. SPXs can be split into three types, based on their chemical structure (Figure 1) [11]. All SPXs are soluble in methanol and chloroform and are therefore readily extracted in lipophilic fractions of shellfish prepared for the mouse bioassay.

SPX A–D were initially isolated and the planar structure of SPX-B and D was described as two lipid-soluble macrocycles containing a spiro-linked tricyclic ether ring system [2]. Following further work, two additional compounds (SPX-E and F) were isolated from the same shellfish extracts and their planar structure was also described. SPX-E and F are thought to be metabolites produced in shellfish as they have so far not been detected in phytoplankton samples from culture or collected in the field and are keto amines without the characteristic heptacyclic iminium ring. Taking into account the absence of activity in the mouse bioassay by i.p. injection of SPX-E or F, it was proposed that the cyclic imine moiety was the spirolide pharmacore [3]. However, following the inactivity in the mouse bioassay (i.p. injection) of SPX-H displaying this function, it was suggested that the cyclic imine moiety is not the only structural requirement for toxicity [17].

The structural elucidation of SPX-A and C was carried out following their isolation from toxic scallops and a culture of toxic *Alexandrium ostenfeldii* [21]. The differences in chemical structures between SPX-C and D, that present an additional methyl substitution on the imine ring, and SPX-A and B may be significant, as the first two compounds are resistant to oxalic hydrolysis whereas the second two compounds are converted to the biologically inactive SPX-E and F when the same reaction is applied [3,20,21].

The identification of SPX derivatives started with the isolation and structure elucidation of 13-desmethyl SPX C present in shellfish extracts and in cultures of *A. ostenfeldii* [21]. Another derivative was identified as 13,19-didesmethyl SPX-C in a culture of *A. ostenfeldii* isolated from Denmark [16]. 20-methyl SPX-G was isolated and its structure elucidated from the digestive gland of steamed Norwegian mussels (*Mytilus edulis*) [22]. The structure of 27-hydroxy-13,19-didesmethyl spirolide C was elucidated from a culture of *A. ostenfeldii* [23] and the last two SPX derivatives, 27-hydroxy-13-desmethyl spirolide C and 27-oxo-13,19-didesmethyl spirolide C, were isolated from the same culture [24].

Following i.p. injection the most toxic SPXs are 13-desmethyl SPX-C, SPX-C and 20-methyl SPX-G, with LD_50_ values of 6.9–8.0 µg/kg b.w. [25]. Mice receiving lethal doses of SPXs died within 3–20 min, with survivors recovering completely [25]. In mice the neurotoxic symptoms described include hunched appearance, abdominal breathing, respiratory distress, contractions, tremors and ultimately death [26].

SPXs are more toxic by the gavage route than when administered on food [9]. By gavage the LD_50_ for the most toxic analogues ranged from 53–176 µg/kg b.w. In feeding trials the LD_50_ values ranged from 500–1005 µg/kg b.w. The signs of toxicity observed were similar to those described following i.p. injection.

### 1.2. Gymnodimines

In New Zealand in 1994, oysters (*Tiostrea chilensis* = *Ostrea chilensis*) analysed by the mouse bioassay for the detection of lipid-soluble marine biotoxins, displayed unusual signs of neurotoxic shellfish poisoning which prompted further investigations. As a result of this research, Gymnodimine (GYM) was isolated and its planar structure determined from dredged oysters from the Foveaux Strait, South Island of New Zealand and from a concurrent bloom of what was thought to be *Gymnodinium* cf. *mikimotoi* [4], but was later renamed as *Karenia selliformis* [27].

**Figure 2:**
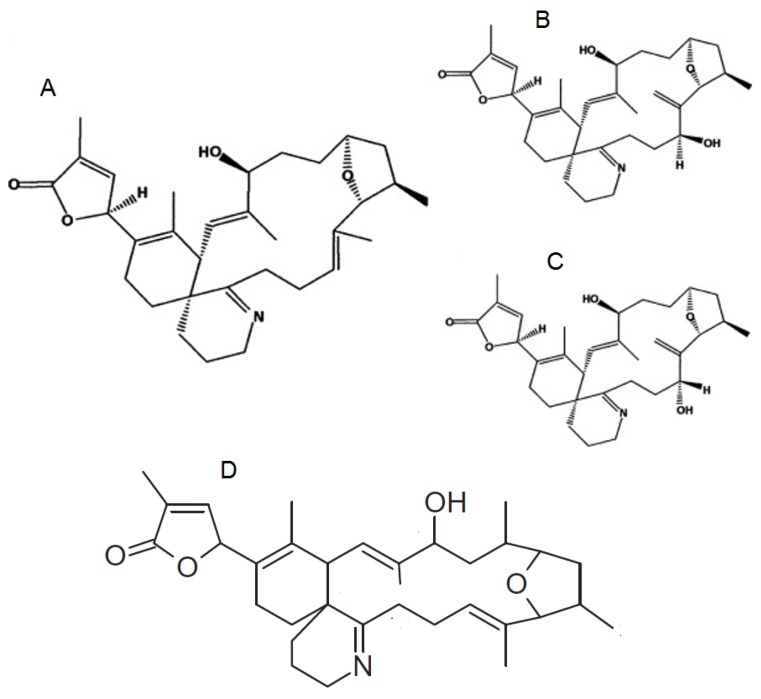
Chemical structure of Gymnodimines (**A**) GYM-A; (**B**) GYM-B; (**C**) GYM-C [31]; and (**D**) 12-methylgymnodimine [32].

The GYMs are the smallest of the cyclic imines and the chemical structure of GYM-A, GYM-B and GYM-C have been elucidated. They include a spirocyclic imine ring and a 16-membered macrocycle (Figure 2) with the absolute stereochemistry of GYM-A elucidated by Stewart (1997) [28]. GYM-B was isolated and its structure identified from a phytoplankton culture [29]. GYM-C was also isolated from extracts of cells and its structure elucidated as a stereoisomer of GYM-B [30].

GYM-A is highly toxic to mice following i.p. injection, with an LD_50_ of 80–96 µg/kg b.w. The signs of toxicity include hyperactivity, jumping, paralysis and extension of the hind legs. Death occurs within 15 min of injection. At sub-lethal doses, prostration and respiratory distress are evident but mice recover completely within 30 min [25]. GYM-A is reported to be 10 times more toxic than GYM-B [33]. The oral LD_50_ for GYM-A by gavage (755 µg/kg b.w.) was eight times higher than i.p. injection in mice. No signs of toxicity were observed in mice administered doses of approximately 7500 µg/kg by feeding [34].

### 1.3. Pinnatoxins

PnTXs are macrocyclic compounds of a 6,7-spiro ring, a 5,6-bicyclo ring and a 6,5,6-trispiro ketal that was initially found accumulated in shellfish of the genus *Pinna*. The causative organism of pinnatoxins in Australia, New Zealand, Japan and France was found to be a peridinoid dinoflagellate [35] identified as *Vulcanodinium rugosum* [36].

**Figure 3:**
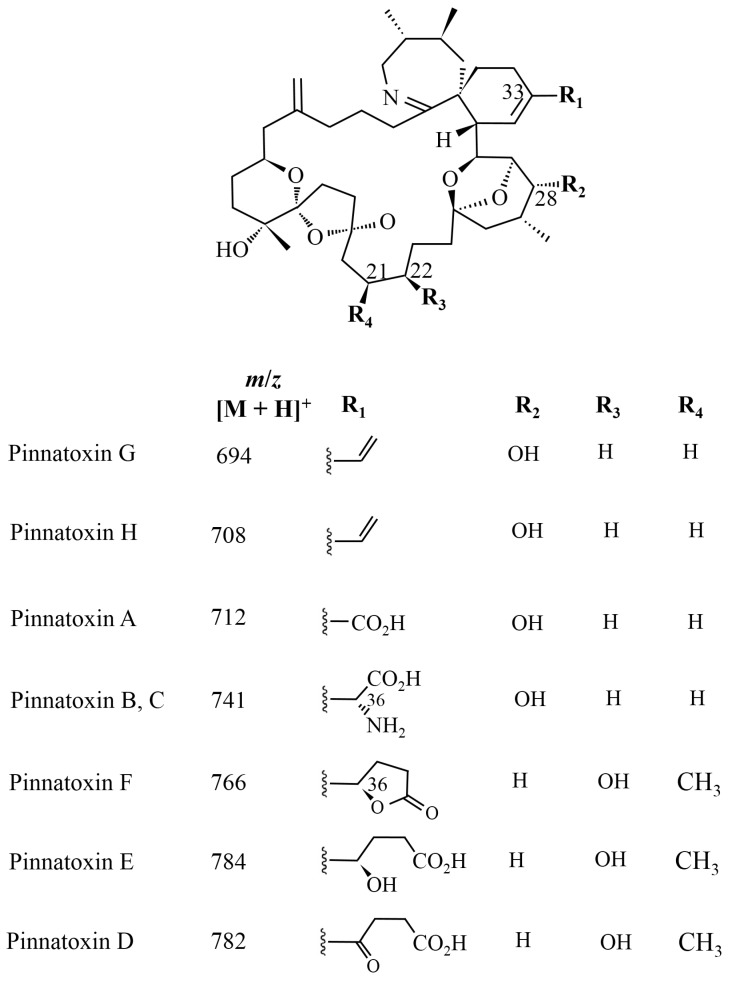
Chemical structure of known pinnatoxins, modified from [40,45].

The chemical structure of PnTXs is closely related to that of the SPXs, with a number of analogues having been identified (A–H) (Figure 3). PnTX-A and B were the first PnTXs isolated from the viscera of Japanese *Pinna muricata* [5]. Subsequently, PnTX-D and C were isolated from the same organism [37,38], with PnTX E, F and G identified from the digestive gland of Pacific oysters (*Crassostrea gigas*) from South Australia [39]. It is now thought that PnTX-F and G are the progenitors of all the known PnTXs (and PtTXs discussed further below) that are produced through metabolism in shellfish [39], with PnTX-F metabolised to PnTX-D and E whilst PnTX-G is metabolised to PnTXs A–C and PtTXs A–C. Most recently PnTX H was identified from a South China Sea *V. rugosum* isolate [40].

The absolute stereochemistry of PnTX-A was established [41] and the total synthesis of the biologically active PnTX-A and of PnTX-G was achieved by Araoz *et al.* (2011) [42]. The absolute stereochemistry of PnTX-B and C was confirmed by total synthesis [43]. Recently, PnTX-H has been isolated from a *V. rugosum* culture [44].

The absolute stereochemistry of PnTX-A was established [41] and the total synthesis of the biologically active PnTX-A and of PnTX-G was achieved by Araoz *et al.* (2011) [42]. The absolute stereochemistry of PnTX-B and C was confirmed by total synthesis [43]. Recently, PnTX-H has been isolated from a *V. rugosum* culture [44].

LD_50_ values for PnTXs range between 16–50 µg/kg b.w., with the analogues E and F being the most toxic [35]. The signs associated with toxicity include hyperactivity, followed by a sudden decrease in activity, abdominal breathing, respiratory failure and death occurring within 22–26 min [6]. At sub-lethal doses, mice showed abdominal breathing and lethargy, but full recovery was evident by 2 h [39].

For PnTX E + F, the LD_50_ following administration by gavage is 23 µg/kg b.w. and 60 µg/kg b.w., respectively following administration in food. The values were estimated from the LD_50_ of algal extract containing 10 µg PnTX/mg [46]. EFSA records that these values are the lowest of any of the cyclic imines [11].

### 1.4. Pteriatoxins

**Figure 4:**
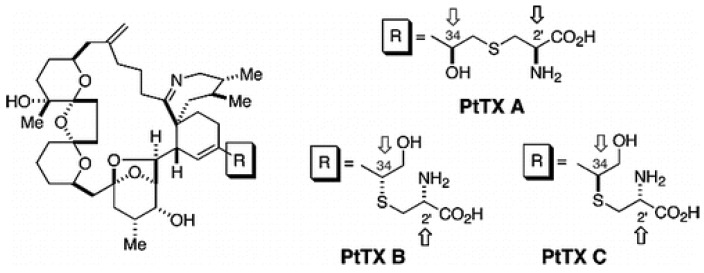
Chemical structure of known pteriatoxins [47].

The chemical structures of the PtTX analogues are similar to PnTXs, but the cyclohexenyl side chain of the PtTXs ends in a cysteine group. PtTXs were isolated from the Okinawan bivalve *Pteria penguin* [6] (Figure 4). PtTX A and a 1:1 mixture of the stereoisomers PtTXs B and C were isolated from the pearl oyster *Pteria penguin* from Japan [6]. Their absolute stereochemistry was confirmed by total synthesis [47]. The origin of PtTXs has not been fully determined but PnTX G may be the precursor of PtTXs A–C via metabolic and hydrolytic transformation in shellfish [39]. The limited data available show that PtTX B/C mix is 12 times more toxic than PtTX A [6].

### 1.5. Prorocentrolides

**Figure 5:**
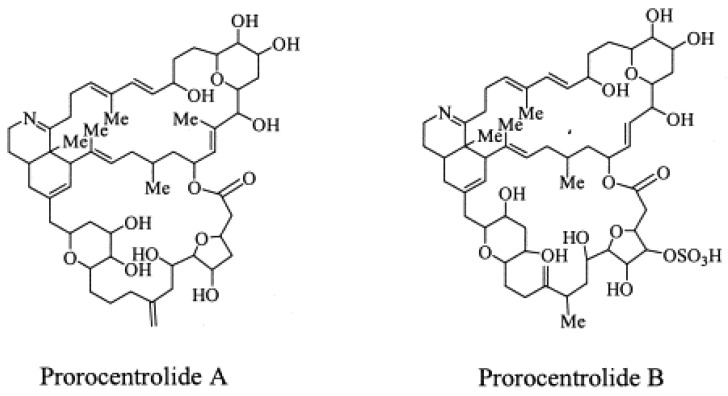
Planar structure of the known prorocentrolides.

PcTX A, an amorphous solid, was first isolated from the epi-benthic dinoflagellate *Prorocentrum lima* from Japan [7] (Figure 5). The planar structure of the toxic macrocycle was elucidated at the same time as PcTX B first isolated from *Prorocentrum maculosum* [3]. The acute toxicity of prorocentrolide has been quoted at 400 µg/kg body weight for lethality following i.p injection in mice [7]. Whilst still described as fast acting toxins, no further information is available regarding toxicity and no adverse effects are reported in humans [48].

### 1.6. Spiro-Prorocentrimine

A polar lipid-soluble macrolide named spiro-prorocentrimine was isolated and purified from a culture of *Prorocentrum* species from Taiwan [9]. The i.p. mouse toxicity was found to result in an LD_99_ of 2.5 mg/kg, hence exhibiting lower toxicity than the other marine cyclic imines. Structural elucidation was conducted using X-ray crystallography and Nuclear Magnetic Resonance (NMR). Similar macrolide structural features were noted in comparison with prorocentrolide [8]. No other toxicity data has been published for this compound [48].

### 1.7. Portimine

A polycyclic ether toxin containing a cyclic imine moiety, was isolated from the marine benthic dinoflagellate *Vulcanodinium rugosum* from New Zealand [10]. The cyclic imine moiety consisted of a five-membered ring with a spiro-link to a cyclohexene ring. The i.p. mouse toxicity LD_50_ was 1570 μg/kg, indicating a much lower toxicity than many other cyclic imine shellfish toxins. However, portimine was highly toxic to mammalian cells *in vitro* with an LC_50_ to P388 cells of 2.7 nM, with activation of caspases indicating apoptotic activity.

## 2. Prevalence of CIs

### 2.1. Spirolides

The presence of SPXs in *Alexandrium* spp. and/or in water samples has been reported in Nova Scotia, Canada [2,3,17,21,49], the Scottish East coast and Orkney Islands [50], Denmark [16], the Gulf of Maine USA [51], Norway [22], Italy [52], Spain [53], and Ireland [15]. The SPX toxin profile produced by *A. ostenfeldii* varies widely between locations, but also with seasonality and even water depth [16,51,54,55,56].

SPXs are present in different species of shellfish, having been reported in mussels (*Mytilus edulis*) and scallops (*Placopecten magellanicus*) harvested from aquaculture sites in Nova Scotia, Canada [2,3,21], in mussels (*Mytilus edulis*) from Norway [22], in mussels (*Mytilus galloprovincialis*) and in razor clams (*Enis arcuatus*) from Spain [57], in clams, mussels and oysters from France [58], in mussels from Italy [13,24] and in macha (*Mesodesma donacium*), clams (*Mulinia edulis*) from Chile [59], pacific oysters (*Crassostrea gigas*) from New Zealand [60] and a range of shellfish from China [61]. In addition, fatty acid acyl esters of SPXs have been detected in Norwegian mussels [18], although these compounds have not been detected in phytoplankton so far, indicating that SPXs might be esterified within the shellfish.

### 2.2. Gymnodimines

In a review of the liquid chromatography mass spectrometry (LC-MS) analysis of 217 shellfish samples collected in New Zealand between 1993 and 1999 and covering eight different species, GYM was detected in 155 of these, with a maximum concentration as high as 23,400 µg/kg [62]. Samples of greenshell mussel (*Perna canaliculus*), blue mussels (*Mytilus galloprovincialis*), dredge oysters (*Tiostrea chilensis*), scallops (*Pecten novaezelandiae*), pipi surf clam (*Paphies australis*), paua or New Zealand abalone (*Haliotis iris*) were all found to be contaminated with GYM, with a wide geographic spread within the country.

The first report of GYM outside of New Zealand was related to clams (*Ruditapes decussatus*) harvested in Tunisia in December 2000 that were analysed using liquid chromatography tandem mass spectrometry (LC-MS/MS). The analysis was carried out on the whole edible meat, on the digestive gland alone and on the remaining edible meat. Gymnodimine (as GYM-A) was detected in all four samples, but the hydroxylated analogues GYM-B and GYM-C were not detected in any of them [63]. GYM was also detected using High Performance Liquid Chromatography (HPLC) with UV detection in clams (*Ruditapes decussatus*) from the coastline of Tunisia that had previously tested positive when assayed by the mouse bioassay [64]. Following testing by LC-MS/MS led the presence of GYM-B and C to also be reported [31].

Following LC-MS/MS analysis, GYM has been detected at low concentrations in seawater, pipis (*Donax deltoides*), mussels (*Modiolus proclivis*) and in oysters (*Saccostrea glomerata*) in Australia [65] in seawater from the Baltic Sea [53] and at low but consistent levels in oysters (*Crassostrea gigas*) from South Africa [66]. The presence of GYM in Canadian waters has also been reported [25] (citing Annual Report 2003–2004 from the Defence Research and Development Canada). Fatty acid acyl esters of GYMs have been detected in shellfish samples from Tunisia [67]. These compounds have not been detected in phytoplankton so far, indicating that the GYMs might be esterified within the shellfish. GYM has also been found in Chinese shellfish [61].

Following consultation with a wide range of UK and international organisations including universities, research institutions, monitoring laboratories and other agencies as part of this review, we received a further report of the presence of GYM on the west coast of Canada but can find no instances of GYM having been detected in European waters to date.

### 2.3. Pinnatoxins

PnTXs reports had initially been limited to the genus *Pinna* in Japan but have now expanded to Pacific oysters (*Crassostrea gigas*) and razor fish (*Pinna bicolor*) from South Australia [39], Pacific oysters from Rangaunu harbour in Northland New Zealand [39,60], mussels from Norway [45,68], mussels from Canada [69], mussels (*Mytilus galloprovincialis*), clams (*Venerupis decussata*) and mussels from France [1,70,71] and seawater, mussels (*M. galloprovincialis*) and Pacific oysters from Spain [72], and from the South China Sea [44]. The extensive survey carried out for this review included a further report of PnTXs in shellfish from Ireland. Concentrations of PnTx reported in shellfish vary widely, but with some authors reporting their occurrence at very high levels. In France, PnTx G was found to reach a maximum of 1.2 mg/kg in mussels [70].

Fatty acid acyl esters of PnTXs have been detected in shellfish including Canadian mussels (*Mytilus edulis*) [73]. These compounds have not been detected in phytoplankton so far, indicating that the PnTXs might be esterified within the shellfish.

### 2.4. Pteriatoxins, Prorocentrolides, Spiro-Prorocentrimine

PtTX-A, B and C were first reported in the bivalve *Pteria penguin* [6] and have not been reported in any other shellfish since. Prorocentrolides and spiro-prorocentrimine were isolated from phytoplankton species [3,7,9] although report of their presence in shellfish has not been found [1].

## 3. Shellfish Accumulation and Depuration

Information related to the shellfish accumulation and depuration of CIs in those sub-groups for which it has been reported is detailed in the individual sub sections below.

### 3.1. Spirolides

The uptake of SPXs in paddle crabs (*Ovalipes catharus*) has been studied using laboratory feeding trials on greenshell mussels (*Perna canaliculus*) as a toxin vector. The toxin uptake in crab was limited to the visceral tissue and the concentrations were low [74]. SPX uptake and detoxification was also investigated in oysters (*Crassostrea gigas*) through laboratory feeding experiments with *A. ostenfeldii*. Four different SPX analogues were detected (13,19-didesmethyl C, 13-desmethyl C, 13-desmethyl D and traces of SPX-D). After 4 days of exposure, the digestive gland of the oysters contained 83% of the total SPX concentration. SPX seemed to have a toxic effect on the digestive tubules of the oysters, but after 7 days depuration in the absence of dinoflagellates, the SPX concentration and the toxic effects had almost completely gone [69].

### 3.2. Gymnodimines

LC-MS/MS analyses of clams collected in Tunisia have shown that GYM accumulates preferentially in the digestive gland, but with a proportion of the total amount also present in the remaining shellfish parts. It is worth noting that the ratio between the amount in the digestive gland and the rest of the shellfish varied from sample to sample [63]. In a separate study carried out on Greenshell mussels collected in New Zealand, GYM was found to be concentrated mainly in tissues outside of the digestive gland and, despite the apparent absence of the producing organism, the concentration did not decrease over the 5-month study [75].

In a kinetic depuration study carried out over 1 month on grooved carpet clams (*Ruditapes dessicatus*) from the coastline of Tunisia, an exponential decrease of 75% of the total GYM-A content during the first 12 days was observed followed by a slow depuration for the subsequent days [64]. In a different study carried out on the same species of clams from Tunisia, faster detoxification rates were observed in the digestive gland when, following exposure, clams were fed a non-toxic algae compared with when they were starved. The detoxification rate was high initially but then decreased and less than 5% remained after 7–8 days [76].

### 3.3. Pinnatoxins

LC-MS analysis of Pacific oysters (*Crassostrea gigas*) and razor fish (*Pinna bicolor*) collected from Franklin Harbour in South Australia revealed that both species were contaminated with PnTXs and that the concentrations were higher in the razor fish than in the oysters [39]. Depuration studies of pinnatoxin E and F produced by New Zealand isolated *V. rugosum* from Pacific oysters were 14.5 and 16 days respectively [77].

## 4. Potential for Cyclic Imines Becoming Established in the UK

Although until recently the majority of the CIs reports were confined to a few locations, the distribution of these has now spread. To date, LC-MS/MS analysis has demonstrated the presence of SPXs as well as PnTXs in UK shellfish (unpublished data). The extensive survey carried out as part of this review received no other reports of CIs compounds in UK waters. The CI-producing phytoplankton threats for UK waters is summarised below.

### 4.1. Alexandrium

The causative organism of SPX production was first identified as *Alexandrium ostenfeldii* [14] from cells isolated from Canadian waters. *A. ostenfeldii* was first described (as *Goniodoma)* from Scandinavian waters with a subsequent re-description by Balech and Tangen (1985) [14]. Its many synonyms are listed by Anderson *et al.* (2012) [78].

*A. ostenfeldii* is broadly distributed in northern temperate latitudes [50], although it is also recorded elsewhere in regions such as Japan, China, U.S.A. and Latin America [53]. In Europe, it has been identified in a number of countries including Ireland [15], Italy [79], Denmark [14] and Scotland [80]. A morphologically similar species, *Alexandrium peruvianum*, found in the Mediterranean Sea [81], in Ireland [15], and in North Carolina [82] has also been confirmed as a producer of spirolides.

Spatial and temporal variability in spirolide profiles exist. Various authors [14,55] finding those from Nova Scotia to often be dominated by 13-desmethyl spirolide C (13-desmeC), but also containing A, B, C or D-type. Similar dominance by 13-desmeC in Mediterranean waters, has also been noted [78]. However, these authors reported that an isolate from the North Sea coast of Scotland yielded exclusively 20-methyl spirolide G (20-meG), and also noted different spirolide profiles from the Celtic Sea, North Sea and Irish coastal waters. Analysis of *A. ostenfeldii* led to identification of five distinct spirolide toxin phenotypes among isolates [51]. It is worth noting that in addition to SPX toxins, some strains of *A. ostenfeldii*, for example from Denmark [78], are also capable of producing paralytic shellfish poisoning (PSP) toxins, with the cause of these differences in toxicity profiles being an active area of research [83].

Potentially biotoxin producing phytoplankton are enumerated within a number of shellfish safety regulatory programmes that are undertaken to fulfil the requirements of EU directives. The methodology used in the UK is explained in detail by Turner *et al.* (2014) [84]. In summary, samples from areas representative of shellfish harvesting sites are collected (weekly in summer and less frequently at other times of the year) and fixed with the preservative Lugol’s iodine prior to enumeration of the potentially harmful cells by the Utermöhl technique [85] following methods accredited by the United Kingdom Accreditation Service (UKAS). The rapid reporting of results required by regulatory monitoring leads to *Alexandrium* counts being reported to genus level only, and hence there is relatively little information on the spatial and temporal distribution of *A. ostenfeldii* (or indeed other *Alexandrium* species) in UK waters.

The genus *Alexandrium* has a complex taxonomic history [78] with numerous species and strains, some of which are known to co-occur [86,87]. While some species are more easily identifiable, *A. ostenfeldii* cells are difficult to discriminate reliably from those of *A. tamarense* using light microscopy alone, as although vegetative cells of *A. ostenfeldii* are typically larger, the size ranges overlap. However, it is occasionally possible to identify individual specimens in Lugols-fixed samples obtained through the monitoring programme. This is done by removal of the cell contents using a dissecting pin, and examination of the thecal plates by phase contrast light microscopy.

Balech (1995) [88] placed both *Alexandrium ostenfeldii* (Paulsen) Balech and Tangen, and *Alexandrium peruvianum* (Balech and Mendiola) Balech and Tangen [89], into the *Alexandrium ostenfeldii* group, based on their morphological similarity. Characteristics that differentiate the two species include cell size, and the shape of the first apical, anterior and posterior sulcal plates. He described *A. peruvianum* as usually being the smaller of the two species, always wider than long, with a curved anterior right margin of the first apical plate, and an irregularly pentagonal-shaped posterior sulcal plate. The anterior and posterior margins of the first apical plate are straighter in *A. ostenfeldii* and form a distinct angle around the ventral pore. The shape of the anterior sulcal plate is also different, more triangular in *A. peruvianum*, and described as similar to a door-latch in *A. ostenfeldii* [82].

Touzet *et al.* (2008) [90] cultured cells from cysts collected from surface sediments around the Irish coast, but found that the morphological features that allow discrimination between vegetative cells of the two species were often variable and shared by both, although plate tabulation patterns were clearly characteristic of *A. ostenfeldii*/*peruvianum*. In an attempt to understand the species boundaries within the *A. ostenfeldii* complex, a comparison of rDNA data, morphometric characters and toxin profiles of cultured isolates from different geographic regions [91] led to the conclusion that the *A. ostenfeldii* complex should be regarded as a single genetically structured species and that *A. peruvianum* should be treated as a synonym of *A. ostenfeldii*.

**Figure 6:**
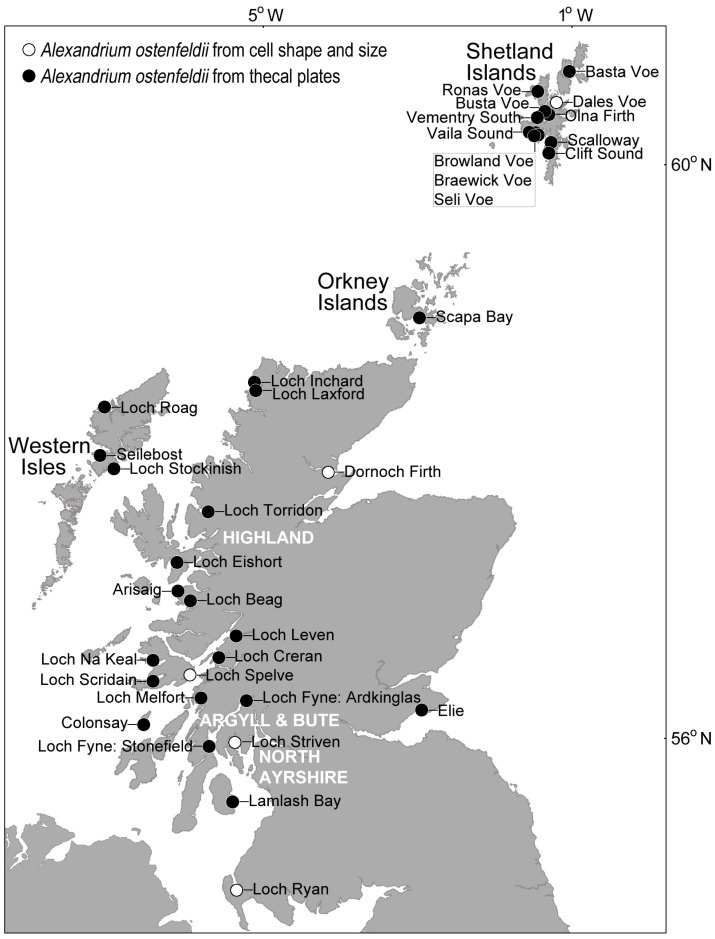
Distribution of cells belonging to the *Alexandrium ostenfeldii* group in Scottish coastal waters. Identification was confirmed at most locations by examination of the thecal plates, but it is likely that the species is much more widespread.

Nevertheless, cells can be assigned to the *Alexandrium ostenfeldii* species complex using the characteristically large ventral pore (described as kidney-shaped [50]) on the first apical plate as the key diagnostic feature. In Scottish waters where UK monitoring is most intensive, the ventral pore readily distinguishes this group from other *Alexandrium* species present, with examination of the thecal plates confirming the presence of *Alexandrium ostenfeldii* group around the Shetland Islands, Orkney Islands, Western Isles, Highland region, Argyll and Bute, and North Ayrshire (Figure 6). Vegetative cells from Scottish coastal waters that were identified as belonging to the *A. ostenfeldii* group based on thecal examination, often exhibited the gross morphological characteristics of this species complex. Cells tended to be large, typically 36–51 µm in width and 33–49 µm in length, and nearly spherical, with a hemispherical hypotheca, convex-conical epitheca and relatively shallow sulcus and cingulum. Using cell shape and size alone, it is likely that *A. ostenfeldii* occurs at many additional sites. Cells tend to be solitary and are typically observed at background concentrations in the water column throughout the year. Cell concentrations rarely exceed 100 cells·L^−1^, and have not been recorded at the bloom densities of 10^5^ to 10^6^ cells·L^−1^ reported in the Northern Baltic Sea [91].

Molecular methods such as fluorescence *in situ* hybridisation (FISH) [50], qPCR, microarrays [91] combined molecular/particle counting approaches [92] offer potential alternative means to discriminate between different *Alexandrium* species, including *A. ostenfeldii*. However, for the present, these remain predominantly research tools.

### 4.2. Other CI Producing Phytoplankton

There are no records of the other known CI producing genera or species in UK waters. The GYM causative species *Karenia selliformis* is restricted to New Zealand and Australia [27]. Its likelihood of appearance in UK waters therefore seems remote. Given the potential for ballast transfer of cells [93] and the similarity of water temperature at least between New Zealand and southern UK waters the potential for survival of cells, should they be trans-located, may exist. Moreover, GYM production by (European strains) of *A. ostenfeldii* has recently been demonstrated [53].

Similarly the causative organism of pinnatoxins (*Vulcanodinium rugosum*) [94] has been identified in Australia, New Zealand and Japan but also in France [36,71]. Moreover, the identification of PnTXs in Norway in 2011 in mussels and in seawater samples [45] as well as temperate Eastern Canadian waters [73], and the South China Sea [40] demonstrates the wider presence of this or another PnTX producing species in European and other waters.

## 5. Toxin Testing Methods

Analytical techniques and their advantages/disadvantages are summarized in Table 1. There have been no reports of human illness linked to CIs [11]. Some seafood poisonings in China and Japan were initially attributed to PnTXs, but these were later shown to be caused by *Vibrio* species [95]. An outbreak of toxicity in Nova Scotia, Canada at a time of high concentrations of SPXs could not be linked to the toxin and clinical signs did not match those observed in mice [96]. Reports from New Zealand indicated that consumers of shellfish with GYMs present suffered no ill effects [34]. Similarly reports from the Rangaunu Harbour region of New Zealand, suggest no ill effects among consumers of shellfish with PnTX concentrations up to 200 µg/kg of shellfish [97].

**Table 1:** Summary of methods applicable to the detection of CI producing phytoplankton and shellfish CIs.

Method	Advantages	Disadvantages
Microscopy	Detection of *Alexandrium* genus Fulfils requirement of legislation	Identification to species too time consuming for routine analysis
Particle counting methods	Potential for more rapid enumeration than by manual cell count	Morphology of causative species prevents easy enumeration Little evidence of suitability for field situations Sample preservation compromises detection
Molecular techniques	Enables species identification	Methods still research based without application to official testing as yet Sample preparation can be time consuming Some required fixatives difficult to use in a monitoring context
Mouse bioassay (MBA)	Direct toxicity assessment in the animal History of use and prevention of sickness	Qualitative Possible interferences No indication of toxin profile Ethical issues Variable performance Not validated
Fluorescence polarization (competitive)	Quick Technically easy Some toxicity assessment (only for the targeted mechanism of action)	No indication of toxicity for toxins with a different mode of action No information on toxic profile Dependent on availability of receptors from *Torpedo marmorata* electric organ Validation data limited Standards required to test performance for other analogues
Fluorescence polarization (direct)	As competitive FP More sensitive than the competitive assay Easier than competitive FP	As competitive assay Tested only in one shellfish species for one SPX
Solid-phase receptor-based assays (RBA)	Simple, sensitive, rapid Good performance in collaborative study Promising fluorescence-based binding assay	Variable affinity for BTX metabolites Requirement for animal tissues and radiolabel Matrix effects Limited development to date with fluorescence-based binding assay
HPLC-UV	Can be automated Quantitative method	Low specificity Standards required for some CIs Not validated
LC-MS(MS)	Can be automated Highly specific Sensitive Toxin profile information available	Expensive instrumentation Lack of availability of all relevant standards No indication of toxicity

### 5.1. Mouse Bioassays (MBA)

SPXs are readily soluble in polar organic solvents and have hence been detected using the MBA developed for lipophilic toxins [98]. The toxins caused rapid death and were initially identified as false positives in the method. The method has also been used to detect GYM-A in clams [63] but the symptoms and time to death are not recorded. Based on the work of Munday [25,99] the method limit of detection for 13 desmethyl SPX-C has been estimated at 5.5 µg/kg and for SPX-C, an LOD of 6.4 µg/kg, whilst the LOD for other spirolides would be significantly higher. Similarly, for GYM-A, the LOD has been estimated at about 77 µg/kg [11]. Based on studies by Selwood *et al.* (2010) [39], the LODs for PnTX-E, F and G are estimated to be 40, 36 and 13 µg/kg [11]. The MBA symptoms are quite specific to the CIs group, with described neurotoxic symptoms including hunched appearance, abdominal breathing, respiratory distress, contractions, tremors and ultimately death [26]. The characteristic neurological response in the MBA shortly after i.p. injection explains the use of the term “fast acting” toxins [100,101]. The MBA provides an indication of the overall CI toxicity of the sample but it does not give information on the individual toxins present. The use of the MBA is highly variable, has not been validated, and is not acceptable in many countries for ethical reasons [11].

### 5.2. Bio-Molecular Methods

#### 5.2.1. Fluorescent Polarization (FP)

A fluorescence polarization assay was developed for analysis of 13-desmethyl SPX-C and GYM-A in shellfish [102]. The assay has been designed as a competitive inhibition assay where the binding of fluorescent α-bungarotoxin, a toxin from snake venom, to the nAChR enriched membrane of the marbled electric ray (*Torpedo marmorata*) is inhibited by GYM and SPXs and is detected by fluorescence polarization. The shellfish (mussels) was extracted using acetone, the solvent evaporated, and a partitioning step using water and hexane was carried out followed by extraction with chloroform. The residue was reconstituted after evaporation and filtered prior to analysis of the resulting extract. The recovery for the extraction method in mussel matrix for GYM-A and 13-desmethyl SPX-C were estimated at 63.6% and 87.4%, respectively. Okadaic acid, yessotoxin and Brevitoxin 2 (BTX-2) were tested for cross-reactivity and did not interfere with the assay. The applicable concentration range in mussels was 50–2000 µg/kg for GYM-A and 70–700 µg/kg for 13-desmethyl SPX-C [102].

Subsequently, the performance of the assay was further tested on four different shellfish matrices: mussels (*Mytilus galloprovincialis)*, clams (*Venerupis semidecussatus*), cockles (*Cerastoderma edule*) and scallops (*Pecten maximus*). The average recovery rates were reported as 90.6% and 89.6% for GYM and 13-desmethyl SPX-C, respectively. The quantitation range in all tested species was 80–2000 µg/kg for GYM and 85–700 µg/kg for 13-desmethyl SPX-C [103]. For mussels and cockles, although the matrix interference was low, the data were statistically different from buffer controls, suggesting that for routine analysis a calibration curve prepared in shellfish matrix may be advisable [103]. In the same study, the variability for 13-desmethyl SPX-C was reported as lower than 14%; however the variability for GYM was greater than 15%. In addition, the detection of 13,19-didesmethyl SPX-C was studied in mussels using the same fluorescence polarization method [104]. The extraction recovery was reported as 77.7% and the quantitation range was 40–200 µg/kg of shellfish meat.

A direct fluorescence polarization assay, involving the labelling of the nAChR from *Torpedo marmorata* with a derivative of fluorescein, was developed for the direct detection and quantitation of SPXs in mussels samples. The assay is based on the change in fluorescence polarization of the labelled nicotinic receptor when bound by a SPX toxin. Three extraction procedures were tested and the extraction procedure taken forward included extraction of the shellfish with methanol and partitioning with dichloromethane followed by evaporation, reconstitution and filtration. The assay was applied to 13-desmethyl SPX-C and 13,19-didesmethyl SPX-C but 13-desmethyl SPX-C was the only one tested in shellfish matrix (mussels). The usable range was reported as 50–350 µg/kg of shellfish meat and the method recovery was 88% [105]. The cross reactivity of the method was tested with two other lipophilic toxins and they were found not to interfere with the assay.

More recently, success with production of a functional microtiter plate receptor binding assay for detection of a wide range of toxins, including cyanotoxins, pinnatoxins, spirolides and gymnodimines was reported [106]. Again, *Torpedo* membranes were used, this time immobilised onto plate surfaces, with biotinylated α-bungarotoxin and streptavidin-horseradish peroxidase facilitating CI detection. The method was tested using 20 PnTx-A spiked shellfish (*Mercenaria mercenaria*) samples from the U.S.A. with a mean PnTx-A assay recovery of 82%. Assay sensitivity was also found to be comparable to previous assays, with an LOD of 1.0 µg/L PnTx-A quoted.

#### 5.2.2. Solid-Phase Receptor-Based Assay

Based on the same principle, another receptor-based method was developed using the competition of 13-desmethyl SPX-C with biotin-labelled α-bungarotoxin, for binding to nAChRs and the immobilisation of the α-bungarotoxin receptor complex on streptavidin-coated surfaces. The quantitation of the immobilized receptor is achieved using a specific anti-nAChR antibody. Three different detection methods have been tested; chemi-luminescent, fluorescent or colorimetric detection [107]. The shellfish (cockles only) was extracted using acetone, the solvent evaporated and a partitioning step using water and hexane was carried out followed by extraction with chloroform. The residue was reconstituted after evaporation and filtered prior to analysis of the resulting extract. The recovery of this extraction method was estimated ~68%. The detection range of the assay for 13-desmethyl SPX-C in cockles was estimated at 40–1000 µg/kg. Although the chemi-luminescent detection offered the best sensitivity (~40 µg/kg in shellfish meat), it appeared to suffer from matrix effects whilst the fluorescence and the colorimetric did not appear to be significantly affected. The assay appears capable of detecting 13-desmethyl SPX-C with a higher sensitivity and a wider dynamic range than the fluorescent polarization assay. The cross-reactivity with other regulated toxins was tested and they were not found to interfere with the results of the assay. In the absence of certified standards, cross reactivity with other CIs has not been tested. The method was also applied to GYM but the detection limit of the method for GYM was 10 times higher than for 13-desmethyl SPX-C [107]. Although this assay is sensitive and specific for the detection of neurotoxins targeting nAChRs, its selectivity is low. In order to address this drawback and provide toxin identification/confirmation, the technique, now termed the *Torpedo* microplate-receptor binding assay, was coupled to mass spectrometry detection [108]. The performance of this assay was tested in four shellfish species; clams (*Glycymeris glycymeris*), oysters (*Ostrea edulis*), mussels (*Mytilus galloprovincialis*) and scallops (*Pecten maximus*) spiked with a mixed standard solution containing GYM-A, 13,19-didesmethyl SPX-C, PnTX-A, 13-desmethyl SPX-C, 20-methyl SPX-G and PnTX-G. The most potent antagonist was 13,19-didesmethyl SPX-C followed by 13-desmethyl SPX-C, PnTX-G, 20-methyl SPX-G, GYM-A and PnTX-A. Cross-reactivity with five other marine toxins was checked and they were not found to interfere [108].

### 5.3. Chemical Methods

#### 5.3.1. HPLC

Most of the CIs lack chromophores, rendering their detection by optical methods quite unspecific. Nevertheless, an HPLC-UV method for the analysis of GYM-A in clams (*Ruditapes decussatus*) from the coastline of Tunisia has been developed. The extraction was carried out on the digestive gland using acetone followed by diethyl ether and dichloromethane. The method recovery exceeded 96% with a limit of quantitation (LOQ) of 8 ng/g of digestive gland [64]. HPLC with ultraviolet detection (HPLC-UV) has also been described for the detection of GYM-A and B in clam tissue [64], with an LOD of 2.4 µg/kg of digestive gland reported. No other methods using HPLC with optical detection exist. For general application to the cyclic imines group, the low UV adsorption of the group means confirmatory analysis is required. No inter-laboratory validation or proficiency test has been undertaken and the lack of certified standards limits development of the approach [11].

#### 5.3.2. LC-MS Methods

The structure of CIs makes them particularly suitable for detection by LC-MS/MS, low detection levels and high specificity can be achieved. LC-MS and LC-MS/MS methods have been widely used for analysis of CIs [6,21,22,38,39,52,57,59,62,63,66,68,75,76,109,110,111,112,113].

The methods are based on reversed-phase LC and electrospray ionization mass spectrometry. They are highly specific (presence of the imino function) and sensitive. Extraction is based on aqueous methanol with optional clean up using either solvent partition or solid phase extraction. Both acidic and alkaline liquid chromatography conditions have been developed to reduce matrix effects. Recoveries of over 90% are reported for SPX-C and GYM-A [112] and LODs of 0.8 and 3.7 pg. on column respectively have been reported [114]. For PnTXs, LODs have not been recorded, however, for PnTX-G the lowest level detected was 5 µg/kg [68]. McNabb *et al.*, (2005) [115] conducted an inter-laboratory study of lipophilic toxin determinations by LC-MS/MS which included the CIs. In this instance only GYM A was quantified, with HORRAT values of 0.8 to 2.0 reported. LC-MS/MS is highly specific and suitable for confirmatory analysis, with LODs lower than other reported methods. However instruments are expensive and require highly skilled operatives and the lack of certified standards is a significant disadvantage [11].

### 5.4. Suitability of Existing and Potential Methods of CI Analysis

CIs are not currently regulated in the EU or in the rest of the world with the information on their toxicity still limited, and mainly confined to acute toxicity studies with a lack of information on any chronic effects. Progress has been made in relation to the mechanisms of action of some CIs (e.g., SPXs, GYMs) but they have not yet been entirely elucidated. As no human toxicity incident has been unequivocally linked to CIs, their inclusion in the list of regulated toxins is still under debate in the scientific community.

LC-MS is the detection method of choice for analysis of CIs in shellfish. However the availability of certified standard commercially has been limited to 13-desmethyl SPX-C, 13,19-didesmethyl SPX-C and GYM-A, with the recent introduction of PnTx-G from the Institute of Biotoxin Metrology within the National Research Council of Canada (NRCC) [116].

13-desmethyl SPX-C and GYM-A are currently the best characterised toxins of the CI group but no inter-laboratory studies have been reported for the validation of these toxins and there is no information related to the validation of an analytical method for the other CIs.

The testing regime currently applied in the UK is not suitable in its current form for the detection of CIs. Whilst the LC-MS/MS lipophilic toxin method has been validated in individual laboratories for GYM-A and 13-desmethyl SPX-C, neither are these toxins reported to the regulators nor are other CIs currently incorporated into monitoring methods. The LC-MS/MS technique would be the most likely candidate since other spirolides and CIs could be added into the monitoring methods if suitable standards were made available.

## 6. Toxicity

Comprehensive reviews of the toxicity of cyclic imines have been undertaken [25,48]. These works summarised the acute toxicities of CIs to animals by both i.p. injection and oral administration for each of the CI classes. The absence of chronic toxicity data for CIs was also highlighted. Also described is the absence of any reports of human illness following consumption of shellfish containing either gymnodimine, pinnatoxins or spirolides, with previously reported instances of PnTx-related poisoning subsequently been shown to relate to the presence of marine pathogens.

### 6.1. Exposure Assessment

SPXs and GYMs are neurotoxic with a similar mechanism of neurotoxicity. Evidence of this is based on inhibition of both mucarinic and nicotinic acetylcholine receptors [25]. The main difference between GYMs and SPXs is that GYMs show a reversible effect whilst SPXs appear to be irreversible [117,118]. Bourne *et al.* (2010) [119] have described the characteristics of SPX and GYM binding to nAChRs including their mechanism of action. Recent work indicated that the toxicity of SPXs is mainly due to their high inhibition potency on various peripheral and central nicotinic AChRs and not to their low ability to interact with muscarinic AChR subtypes [42,120]. PnTXs cause respiratory paralysis in mice and it is thought they also target nAChR [39,42]. There are currently no data on the mode of action of PtTXs.

EFSA determined consumption of 400 g of shellfish in one meal is typically taken to represent a large portion size [11]. As CIs are not regulated, there is the potential for contaminated produce to reach the consumer. EFSA estimated that based on the 95th percentile figure of 8.9 µg/kg, consumption of 400 g would result in a single exposure of 0.06 µg/kg b.w. or 3.6 µg per person. EFSA determined a probabilistic estimate of dietary exposure based on the distribution of both the occurrence data and data on shellfish consumption. The chance of exceeding the exposure estimate of 3.6 µg per person corresponding to a portion of 400 g containing the 95th percentile concentration (3.4 µg per portion) was estimated to be 4% [11].

### 6.2. Toxicokinetics

EFSA reported that there is no information on the absorption, distribution or excretion of SPXs, GYMs or PnTXs in laboratory animals or humans [11]. Information based on oral administration [25,34,96] indicate that the compounds are absorbed from the intestinal tract including in humans from where they have the ability to reach different organs [121]. Analogues of SPX-C can be detected in the blood, urine and faeces of mice following oral administration [11,101], and in the brain following i.p. injection [122] Rapid recovery following sub-lethal doses of GYM-A or 13-desmethyl SPX-C [34] suggests rapid detoxification or excretion. The EFSA panel report that there is no information on the biotransformation of CIs in mammals but it is known that biotransformation and detoxification occurs in molluscs [3,25,28]. Munday (2014) more recently confirmed the absence of published information relating to the metabolism and disposition of CIs in animals, with the exception of 13-desmethyl spirolide C and 13,19-didesmethyl spirolides C [48]. Following oral administration these compounds were absorbed quickly, with rapid removal from blood and detectable presence in urine and faeces at 1 h and 24 h post-feeding respectively [64].

### 6.3. Relative Potency of Analogues

EFSA report that the cyclic imines appear to act through the blocking of AChR receptors [11]. These interactions may differ between the groups, and hence both reversible and irreversible effects are seen. Hence, whilst there are no data on combined exposure, it is reasonable to assume additive toxicity by the different analogues within each group. In Europe, only a limited number of CIs have been detected and hence the current practice of reporting the concentration as the sum of the analogues, with a factor of one for each analogue is justified.

There are no data available on long term studies to establish a tolerable daily intake for the CIs. The EFSA Panel noted that oral toxicity varies depending on the method of administration, gavage or in feed [11]. Whilst there are no quantitative data on human toxicity, the Panel were of the opinion that given their acute toxicity, acute reference doses should be established for the different groups. However the Panel indicated that there are insufficient data on which to establish ARfDs.

EFSA report that data reported on oral toxicity in mice was not appropriate for the establishment of an ARfD [11]. They did however calculate a margin of exposure (MOE) based on the oral LD_50_ and the 95th percentile of exposure (0.06 µg/kg b.w.). The MOE ranges from 1000 to 10,000 depending on whether the LD_50_ from gavage or in feed is used. The Panel felt that the LD_50_ from administration in food was the most appropriate. Based on their analysis, they concluded that exposure to SPXs does not raise concern for the consumer. They note however that there is a high degree of uncertainty associated with estimating exposure for the European population due to the lack of occurrence data.

## 7. Conclusions: Summary of Current and Future CI Threats and Options for the Monitoring of Toxins and Their Causative Organisms to Meet Legal Requirements

Current UK and other regulatory biotoxin producing phytoplankton microscopy based monitoring programmes identify and enumerate biotoxin producing phytoplankton to genus only. CI producing *A. ostenfeldii* are known to exist in UK waters, but these co-exist with STX producing and benign species, and current monitoring practices do not allow their routine discrimination. As other CI producing genera are not known to be present, they are not monitored. However, given that PnTXs have been identified in Norway and the water conditions in the UK are not dissimilar to those in New Zealand where *K. selliformis* generated GYMs occur, the risk of other (perhaps ballast transferred) CIs in UK waters cannot be discounted, particularly in a changing climate. A molecular based monitoring approach for unusual species applied on a precautionary basis at key sentinel sites co-incident with major shellfisheries and/or major ports would potentially be of value in reducing risk and better evaluating the distribution of CI producing organisms. SPXs have been identified in the UK and a number of other European countries and whilst GYMs have not been formally reported.

The concentration of CIs in shellfish is not regulated in the European Union or in other parts of the world. This is consistent with the lack of definitive reports of human illness due to SPXs, GYMs, PnTXs or PtTXs. Yet given the detection of CIs by MBA, their potential for negatively impacting human health cannot be discounted and a guidance level of 400 µg/kg of SPXs/kg of shellfish has been recommended by the EURL working group on toxicology. At this level The CONTAM Panel concluded that current estimated exposure to SPXs does not raise concern for the health of the consumer, although this conclusion is based on very limited toxicity data [11]. Exposure to GYMs, PnTXs and PtTXs could not be estimated from the available data therefore no conclusions could be drawn with respect to any possible risk to consumers.

Addressing the knowledge gaps that exist with regards CIs toxicity and biogeography would be beneficial to the UK shellfish industry that carries the legal responsibility for the safety of the produce it sells. More detailed toxicological understanding of CIs is required, in particular related to any chronic effect and potential synergistic effects with other marine biotoxins. Acyl-esters of CIs have been identified in shellfish so their bio-availability to human through shellfish consumption needs to be investigated.

Receptor-based and analytical methods exist that would be of value in the quantification of CIs. Development of an ethical screening assay directly related to toxicity with application of a suitable confirmatory method for quantitation such as an LC-MS method providing high specificity and toxin profile would benefit understanding and minimise industry risk. However, such an approach is subject to further development including validation of methods, together with the purification and commercial provision of certified standards. In order to meet legal requirements associated with official control monitoring of bivalve molluscs, each of these assays would need to undergo a series of validation studies to determine full performance characteristics of the method. In addition, the performance would need to be demonstrated as being able to provide at least the same level of effectiveness as the MBA.
